# Quantification, Antioxidant and Antimicrobial Activity of Phenolics Isolated from Different Extracts of *Capsicum frutescens* (Pimenta Malagueta)

**DOI:** 10.3390/molecules19045434

**Published:** 2014-04-24

**Authors:** Patrícia L. A. Nascimento, Talita C. E. S. Nascimento, Natália S. M. Ramos, Girliane R. Silva, José Erick Galindo Gomes, Rosângela E. A. Falcão, Keila A. Moreira, Ana L. F. Porto, Tania M. S. Silva

**Affiliations:** 1 Department of Morphology and Animal Physiology, Federal Rural University of Pernambuco, Av. Dom Manoel de Medeiros, s/n., 52171-900 Recife, Pernambuco, Brazil; E-Mails: talitacamila07@gmail.com (T.C.E.S.N.); erick.galindo.zoo@hotmail.com (J.E.G.G.); analuporto@yahoo.com.br (A.L.F.P.); 2 Department of Molecular Sciences, Federal Rural University of Pernambuco, Av. Dom Manoel de Medeiros, s/n., 52171-900 Recife, Pernambuco, Brazil; E-Mails: quinathi@gmail.com (N.S.M.R.); girlianeregina@yahoo.com.br (G.R.S.); taniasarmento@dcm.ufrpe.br (T.M.S.S.); 3 Department of Biological Sciences, University of Pernambuco, Rua Capitão Pedro Rodrigues, 105, 55290-000 Garanhuns, Pernambuco, Brazil; E-Mail: rosangela.falcao@terra.com.br; 4 Academic Unit of Garanhuns, Federal Rural University of Pernambuco, Av. Bom Pastor, s/n, 55292-270 Garanhuns, Pernambuco, Brazil; E-Mail: moreiralab@yahoo.com.br

**Keywords:** *Capsicum frutescens*, antioxidant, antimicrobial, chrysoeriol, capsaicin, dihydrocapsaicin

## Abstract

This paper presents the quantification, antioxidant and antimicrobial activity of capsaicin, dihydrocapsaicin and the flavonoid chrysoeriol isolated from different extracts (hexane and acetonitrile extracts from whole fruit, peel and seed) of *Capsicum frutescens* (pimenta malagueta). The acetonitrile extract of the seeds, peel and whole fruits contained capsaicin as a major component, followed in abundance by dihydrocapsaicin and chrysoeriol. The antimicrobial activity of the isolated compounds against seven microorganisms showed chrysoeriol was the most active compound. In the antioxidant test, the acetonitrile extract from the whole fruit showed the highest activity. The antioxidant activity of pimenta malagueta may be correlated with its phenolic content, principally with the most active compound, capsaicin.

## 1. Introduction

The genus *Capsicum* of the Solanaceae family has high economic value due to its use as a food and it is endemic in the tropics, particularly in Central America and South America [1]. In Brazil there are a large number of species [2], and one of these species, commonly known as “pimenta malagueta”, is widely consumed.

Capsaicin (8-methyl-*N*-vanillyl-6-nonenamide) is the main compound in the genus, and together with a group of similar substances called capsaicinoids, which includes dihydrocapsaicin and nordihydrocapsaicin, it is responsible for over 90% of *Capsicum* fruit pungency [3,4].

Peppers contain phenolic compounds, flavonoids and carotenoids, besides being a source of vitamin C [5]. Among these, flavonoids are ubiquitous phytochemicals found in plants with a wide group of exploitable activities, including antimicrobial activity, antibiotic synergism and bacterial virulence removal [6]. Once absorbed, they influence several biological functions, including protein synthesis, angiogenesis, cell proliferation and differentiation, thus benefiting a variety of human diseases [7]. The flavonoids found in most peppers are glycosides and aglycones of myricetin, quercetin, luteolin, apigenin and kaempferol [8].

Concerning their biofunctional activities, capsaicinoids produce physiological and pharmacological actions, and show anticancer effects, act against high cholesterol levels and obesity and are used to treat arthritis pain [9]. Capsaicin also possesses antimicrobial properties, which suggests its use as a potential natural inhibitor of pathogenic microorganisms in food [10].

The antioxidative and antimicrobial properties of many plant extracts are of great interest as natural additives in both academia and the food industry because there is a growing tendency to replace synthetic antioxidants with natural ones [11]. Oxidative stress plays an important role in various diseases that show a high worldwide prevalence, such as cancer, rheumatoid arthritis, asthma, diabetes, cardiovascular and neurodegenerative diseases, including atherosclerosis, Alzheimer’s disease, and other age-related degenerative disorders [12]. 

Many studies on the Capsicum genus have been reported [13,14,15], including *Capsicum* *frutescens* [16,17,18,19]. However in the studies with *Capsicum* *frutescens* there are no details correlating the quantification of capsaicinoids and flavonoids in the various parts that compose the fruit with extraction using different solvents.

With the objective to investigate which part of the fruit of pimenta malagueta concentrated the major quantities of the principal phenolic compounds, in this study, we conducted a quantification of capsaicin, dihydrocapsaicin and chrysoeriol by HPLC-DAD, isolated in hexane and acetonitrile extracts of whole fruits, peel and seeds of *Capsicum frutescens*. The total phenolic contents and the antioxidant properties of extracts and compounds were also studied. In addition, the antimicrobial activity of the isolated compounds was tested against seven microorganisms. 

## 2. Results and Discussion

### 2.1. Phytochemical Content

**Figure 1:**
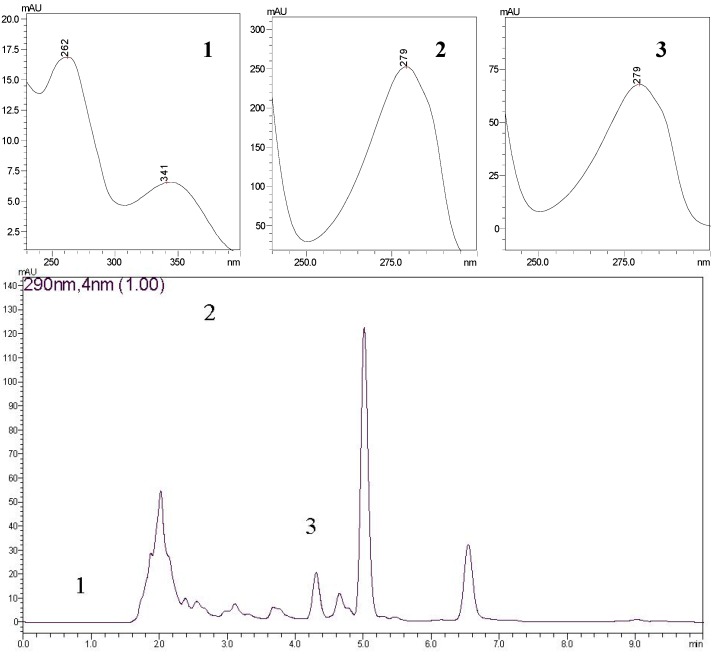
Chromatogram (HPLC-DAD) for an acetonitrile extract of *C. frutescens* whole fruits. Chrysoeriol (**1**); capsaicin (**2**); and dihydrocapsaicin (**3**).

The phenolic, flavonoid and capsaicinoid contents in the hexane and acetonitrile extracts of seeds, peel and whole fruits of *C. frutescens* are reported in Table 1. The phenolic content ranged from 3.2 ± 0.22 to 110.6 ± 1.03 mg GAE g−1 of extract (milligrams of gallic acid equivalent per gram). Our data showed the highest phenolic content in acetonitrile extract of whole fruits. In contrast, Howard et al. [20] and Zhuang et al. [21] found 5.1 and 4.9 mg GAE g−1 for *C. frutescens* fruits, respectively, which might be due to the different cultivars studied and growing conditions used. Among the parts of the fruits, the seeds contained 61.31 ± 0.64 mg GAE g−1, whereas the peel contained 14.0 ± 0.14 mg GAE g−1 in an acetonitrile extract. Oboh and Ogunruko [22] noted that there is a higher phenolic content in the pericarp of the fruits of *C. frutescens* when compared with the seeds, which is in contrast with our findings. Therefore, our results suggest that acetonitrile was able to extract a higher proportion of phenolic compounds from *C. frutescens* than hexane and ethanol [19]. The chromatogram for an acetonitrile extract of *Capsicum frutescens* whole fruits is shown in Figure 1.

**Table 1:** Phytochemical content at hexane and acetonitrile extracts of seeds, peel and whole fruits of *C. frutescens.*

Plant part	Solvent	Phenolics (mg GAE g^−1^ of extract)	Capsaicin (mg g^−1^ extract ± SD)	Dihydrocapsaicin (mg g^−1^ extract ± SD)	Chrysoeriol (mg g^−1^ extract ± SD)
**Seeds**	**Hexane**	9.4 ± 0.55	90.0 ± 8.60	42.0 ± 4.21	1.8 ± 0.69
	**Acetonitrile**	61.3 ± 0.64	130.4 ± 4.98	53.3 ± 1.59	11.4 ± 0.38
**Peel**	**Hexane**	3.2 ± 0.22	45.8 ± 0.1	23.3 ± 0.23	0.5 ± 0.27
	**Acetonitrile**	14.0 ± 0.14	164.3 ± 10.84	73.6 ± 6.43	5.1 ± 1.31
**Whole Fruits**	**Hexane**	4.9 ± 0.44	31.1 ± 2.04	15.0 ± 0.65	0.4 ± 0.03
	**Acetonitrile**	110.6 ± 1.03	109.8 ± 13.66	42.0 ± 4.64	5.5 ± 0.49

Chinn *et al.* [23] noted that the choice of solvent should be made according to the degree of solubility of the pigments present and this is a major factor that influences the molecule purification process. Tapia *et al.* [24] related that solid-liquid extraction with solvents such as hexane is the most commonly employed method for capsaicinoid recovery.

In this study, we initially used hexane to remove the oil portion due its low polarity and then used acetonitrile to easily extract the phytochemical compounds and thus isolated 1,184 mg of capsaicin, 778 mg of dihydrocapsaicin and 36 mg of chrysoeriol. The dihydrocapsaicin and chrysoeriol isolated in the HPLC were used as standards for quantification. Bae *et al.* [8] extracted *C. annuum* with hexane and obtained maximum amounts of capsaicin and dihydrocapsaicin, ranging from 0.03 to 2.4 mg g^−1^ and 0.01 to 1.0 mg g^−1^, respectively. These values are lower than those shown in Table 1.

During the quantitative analysis of capsaicin, dihydrocapsaicin and chrysoeriol content by HPLC, acetonitrile yielded the highest amount of the compounds (Table 1). These findings agree with a previous study performed by Collins et al. [25]. The acetonitrile extracts of peel showed the highest concentrations of capsaicin (164.3 ± 10.84 mg g−1 extract) among the parts assessed. Several authors have evaluated the capsaicin and dihydrocapsaicin content of peppers of the genus *Capsicum*, in addition to their different species, using various techniques and solvents for extraction of chemical constituents. The contents found in this study were in greater abundance than those reported in a previous study (Table 2). The main factors which determine the concentration of capsaicin in certain species are related to light intensity and culture temperature as well as the age and position of the fruit on the plant [9].

Cisneros-Pineda *et al.* [4] noted that the biosynthesis of capsaicinoids occurs in the placenta, where the specialised epidermal cells accumulate in vacuoles and excrete on the inner surface of the seed and pericarp; therefore, the capsaicinoids should accumulate preferentially in the placenta rather than in the pericarp, which differs from our results showing higher concentrations in the peel. A likely explanation for our findings is that the presence of capsaicinoids in the pericarp suggests that capsaicinoids are translocated from the placenta to the pericarp tissue via the cell walls of the epidermal layer of the placenta [26]. 

Capsaicinoids were not evenly distributed in the fruit. Perhaps the lowest capsaicin content recovered from the whole fruits was due the decrease in capsaicinoids when the cells were disrupted [27]. While separating the seeds from fruit, the placenta was collected with the peel. This justifies the higher levels of capsaicinoids in this part. The seeds had the second highest level (130.4 mg g^−1^), probably due to the proximity of the structures responsible for the synthesis of capsaicinoids with the seeds [23].

**Table 2:** Capsaicin and dihydrocapsaicin content in various species of *Capsicum*.

Species/part	Extraction solvent	Capsaicin	Dihydrocapsaicin	[Reference]
*C. frutescens*	Ethanol	0.7 mg g^−1^ fresh weight	0.4 mg g^−1^ fresh weight	Zhuang *et al.*, 2012 [21]
*C. annuum*	Methanol	2.3 mg g^−1^ extract	0.8 mg g^−1^ extract	Alvarez-Parrilla *et al.*, 2011 [5]
*C. annuum*	Ethanol	0.3 mg g^−1^ extract	0.2 mg g^−1^ extract	Othman *et al.*, 2011 [9]
*C. chinense* Seeds	Acetonitrile	14.0 mg g^−1^ dried fruits	5.2 mg g^−1^ dried fruits	Chinn *et al.*, 2011[23]
*C. chinense* Shells		5.0 mg g^−1^ dried fruits	1.3 mg g^−1^ dried fruits	
*C. chinense* Whole fruits		7.0 mg g^−1^ dried fruits	2.0 mg g^−1^ dried fruits	
*C. frutescens* Seeds	Methanol	1.1 mg g^−1^ fresh weight	0.5 mg g^−1^ fresh weight	Wahyuni *et al.*, 2011 [26]
*C. frutescens* Pericarp		0.6 mg g^−1^ fresh weight	0.1 mg g^−1^ fresh weight	
*C. chinense*	Ethanol	4.3 mg g^−1^ fresh weight	2.4 mg g^−1^ fresh weight	Menichini *et al.*, 2009 [1]
*C. frutescens*	Acetonitrile	3.7 mg g^−1^ dried weight	2.4 mg g^−1^ dried weight	Garcés-Claver *et al.*, 2006 [3]

Mature *C. annuum* and *C. frutescens* cultivars are appreciably higher in total flavonoids than *C. chinense* cultivars at the maturity stage [20]. Marín *et al.* [28] showed that the phenolic compounds in sweet peppers were mainly located in the peel. They characterised 23 flavonoids from the pericarp of sweet pepper, including chrysoeriol. In the present study, chrysoeriol content was higher in the acetonitrile extract of seeds (11.4 mg g^−1^) than the acetonitrile extract of peel. Howard *et al.* [20] and Zhuang *et al.* [21] analysed the luteolin concentration in *C. frutescens* and found 0.03 mg g^−1^ and 0.8 µg g^−1^ luteolin, respectively. On the other hand, the lowest concentration of our study was in the hexane extract of whole fruits (0.4 ± 0.03 mg g^−1^).

Peterson and Dwyer [7] proposed a botanical classification scale for flavonoid concentration that rates foods as low (0.1–39.9 mg kg^−1^), moderate (40–99.9 mg kg^−1^), and high (>100 mg kg^−1^). From this scale, our findings showed a high concentration of chrysoeriol for all of the samples tested (0.4–11.4 mg g^−1^ extract). However, the quantitative variation of pepper flavonoids occurs based on the different extraction procedures, such as extraction solvent, sample to solvent ratio, and extraction time [8]. 

### 2.2. Radical Scavenging and Antioxidant Activity

The antioxidant assays can be classified into two categories: tests that measure the ability to scavenge free radicals and trials that assess the ability to inhibit lipid oxidation, with the ability to eliminate free radicals being one of the mechanisms that contribute to the overall activity providing a synergistic effect [11].

**Figure 2:**
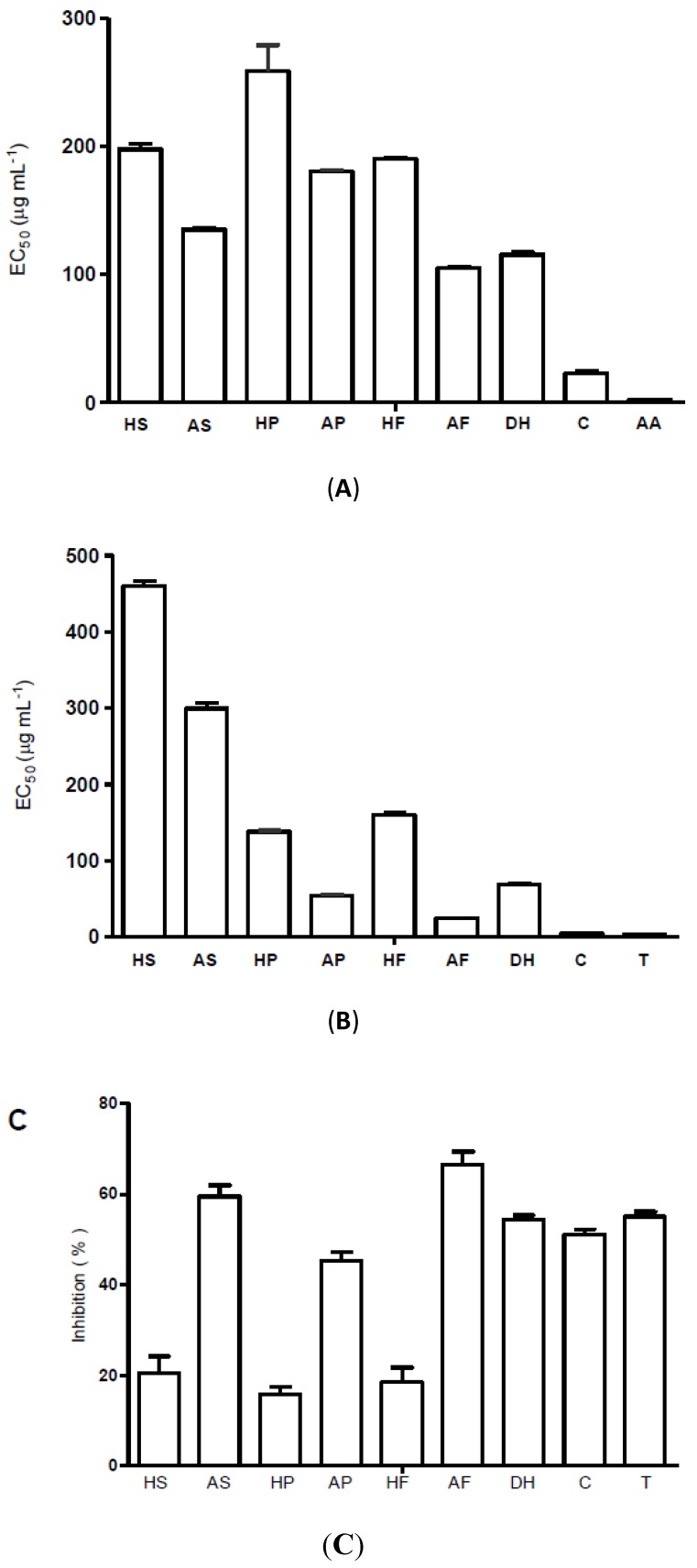
Effects of hexane and acetonitrile extracts of *C. frutescens*, capsaicin and dihydrocapsaicin on DPPH radical (**A**), ABTS radical; (**B**) and β-carotene/linoleic acid assay at 60 min; (**C**). HS: hexane seeds, AS: acetonitrile seeds, HP: hexane peel, AP: acetonitrile peel, HF: hexane whole fruits, AF: acetonitrile whole fruits, DH: dihydrocapsaicin, C: capsaicin, AA: ascorbic acid and T: Trolox. The confidence interval was 95% (*p* < 0.05).

A method commonly used to verify the antioxidant ability consists of measuring the ability of extracts or pure molecules to scavenge DPPH^•^. Antioxidants are able to reduce the DPPH^•^ from purple colour to yellow colour upon receiving an electron or a hydrogen radical [29]. The radical scavenging effects and antioxidant activity are demonstrated in Figure 2. Acetonitrile extracts showed the lowest EC_50_ for the DPPH and ABTS assays, even though they showed better antioxidant activity when compared to hexane extracts. When the non-polar constituents were selected by extraction with hexane, the activity was reduced.

In this study, all of the samples were able to reduce the stable free radical 2,2-diphenyl-1-picrylhydrazyl (DPPH) to the yellow-coloured diphenylpicrylhydrazine, with EC_50_ values ranging from 23.1 to 258.6 µg mL^−1^. Capsaicin showed the lowest EC_50_ value among the samples (23.1 µg mL^−1^), and Tukey’s test showed no statistically significant difference between capsaicin and ascorbic acid (*p* > 0.05). Zhuang *et al.* [14] studied the DPPH scavenging activity of *C. frutescens* fruits and found an EC_50_ of 135.1 µg mL^−1^, higher than our best result for whole fruit (105.1 µg mL^−1^). Menichini *et al.* [1] studied fruits of *C. chinense* and found an EC_50_ of 287 µg mL^−1^, and Zimmer *et al.* [12] analysed seeds of *C. baccatum* and found an EC_50_ ranging from 229.72 to 819.67 µg mL^−1^; both are higher values than those found in this study. The best EC_50_ of the samples tested in our study was capsaicin (23.1 µg mL^−1^); it was the closest value to ascorbic acid and to the Zimmer *et al.* [12] result, which showed an EC_50_ of 17.62 µg mL^−1^. 

The free radical scavenging activity on ABTS showed EC_50_ values ranging between 24.3 and 459.6 µg mL^−1^ (Figure 1). The extract that showed the lowest EC_50_ value was the acetonitrile extract of fruit, followed by acetonitrile extract of peel (53.3 µg mL^−1^). Capsaicin had the best effective concentration of the samples tested (3.9 µg mL^−1^). Moreover, it may be noteworthy that Tukey’s test showed no statistically significant difference between the results of capsaicin and Trolox (*p* > 0.05), which highlights the excellent free radical scavenging action of capsaicin.

The *β*-carotene/linoleic acid test evaluates the inhibitory effect of a compound or a mixture on the oxidation of *β*-carotene in the presence of molecular oxygen (O_2_). An assay of the remaining *β*-carotene provides an estimation of the antioxidant potential of the sample. An antioxidant is a substance that significantly prevents or delays the oxidation of an oxidisable substrate when present in a low concentration [29].

As shown in Figure 2, the antioxidant activity at 60 min, half the total time of the test, showed inhibitory activity ranging between 15.4% and 66.4%. The acetonitrile extracts of seed and whole fruits showed the best results (59.4% and 66.4%), higher than the standard Trolox (55.0% ± 1.09%). Our results for capsaicin (51.1% ± 1.18%) and dihydrocapsaicin (54.2% ± 1.07%) did not differ statistically from those with Trolox (*p* > 0.05). As compared to the total phenolic content (TPC), the antioxidant activity and radical scavenging expressed by EC_50_ showed the same tendency. The highest value in TPC had the strongest radical scavenging activity and better antioxidant activity among the extracts. The higher antioxidant activity (66.4%) and lowest DPPH (105.1 µg mL^−1^) and ABTS (24.3 µg mL^−1^) EC_50_ of the acetonitrile extract of whole fruits corresponds to the highest value of TPC (110.6 mg AGE.g^−1^).

It was pertinent to perform a linear correlation between the values of total phenolic content depending on the values of DPPH, ABTS and antioxidant activity with the *β*-carotene/linoleic acid system. The Pearson correlation coefficient indicates that 94% of the scavenging activity displayed by the extracts against the radical ABTS^•+^ (r = 0.9445) and DPPH^•^ (r = 0.9439) and 88% of total antioxidant activity (r = 0.8863) can be attributed to the phenolic content present in these samples. According to Chinn *et al.* [23], the processing conditions used for the capsaicinoid extraction influences the quality and antioxidant potential of these compounds.

### 2.3. Antimicrobial Activity

Antimicrobial activity of capsaicin, dihydrocapsaicin and chrysoeriol isolated from the acetonitrile extract of *C. frutescens* was tested against seven microorganisms. These were three Gram negative bacteria (*E. coli*, *P. aeruginosa*, and *K. pneumoniae*), three Gram positive bacteria (*E. faecalis*, *B. subtilis*, and *S. aureus*) and one yeast (*C. albicans*). The results of the minimal inhibitory concentration determination for the microorganisms tested are presented in Table 3. It can be observed that the MIC values obtained confirm the existence of significant activity against the bacterial strains tested in our study, with MIC values ranging between 0.06 and 25 µg mL^−1^. Our results show that Gram positive and Gram negative microorganisms were affected by the three compounds tested. The minimal inhibitory concentration for chrysoeriol to inhibit the yeast growth was not detected.

**Table 3:** Minimal inhibitory concentration (MIC) of capsaicin, dihydrocapsaicin and chrysoeriol to seven microorganisms.

Microorganisms	MIC (µg mL^−1^)		
	Capsaicin	Dihydrocapsaicin	Chrysoeriol
**Gram positive bacteria**			
*Enterococcus faecalis*	25	0.6	1
*Bacillus subtillis*	25	1.2	1
*Staphylococcus aureus*	1.2	5	0.25
**Gram negative bacteria**			
*Pseudomonas aeruginosa*	10	2.5	0.12
*Klebsiella pneumoniae*	0.6	2.5	0.25
*Escherichia coli*	5	5	0.06
**Yeast**			
*Candida albicans*	25	10	ND *

* ND: Not detected.

Dihydrocapsaicin required higher concentrations to inhibit Gram negative bacteria (2.5–5 µg mL^−1^) than to inhibit Gram positive bacteria (0.6–5 µg mL^−1^); this result is justified because the Gram positive bacteria are expected to be more susceptible due to having only an outer peptidoglycan layer, which is not an effective permeability barrier [29]. Concerning the capsaicin-related MIC, the high concentrations needed to inhibit *E. faecalis*, *B. subtilis* and *P. aeruginosa* may be related to the fact that some bacteria use capsaicin as a nutrient for growth [30]. Therefore, our results show that dihydrocapsaicin possesses a selective antimicrobial activity on the basis of the cell wall differences in bacteria. In this study, the growth of *Candida albicans* was inhibited for capsaicin and dihydrocapsaicin, emphasising that dihydrocapsaicin showed the lower MIC (10 µg mL^−1^). A previous study performed by Molina-Torres *et al.* [31] required a MIC of 300 µg mL^−1^ and 25 µg mL^−1^ to inhibit *E. coli* and *B. subtilis* growth, respectively, with capsaicin. Our findings corroborate the results of the previous study for *B. subtilis* but disagree regarding the observations for *E. coli*. Contradicting our results, Dorantes *et al.* [10] did not find any antimicrobial activity for capsaicin and dihydrocapsaicin against *B. subtilis,* *S. aureus* and *C. albicans*.

Interestingly, our current findings show a remarkable antimicrobial activity against Gram negative bacteria ranging from 0.06 to 10 µg mL^−1^. To put these values into context, extracts with MICs ≤ 100 µg mL^−1^ and isolated compounds with MICs ≤ 10 µg mL^−1^ are considered to be very interesting [32]. Recent studies have identified some flavonoids with MICs as low as 0.06 µg mL^−1^ [6]. It should be noted that among the isolated compounds tested, chrysoeriol showed the lowest MIC values. Interestingly, our results for this compound show remarkable activity against Gram-negative bacteria.

## 3. Experimental

### 3.1. Chemicals and Solvents

All solvents used were of commercial HPLC grade. The β-carotene, 2,2-diphenyl-1-picrylhydrazyl (DPPH), linoleic acid, 6-hydroxy-2,5,7,8-tetramethylchroman-2-carboxylic acid (Trolox), potassium persulfate, 2,2'-azinobis-(3-ethylbenzothiazoline-6-sulfonic acid) (ABTS), gallic acid, and the Folin-Ciocalteu reagent were purchased from Sigma–Aldrich (St. Louis, MO, USA). 

### 3.2. Preparation of Extracts from Capsicum frutescens

*Capsicum frutescens* fruits (1 kg) were dried in a circulating air oven (50 °C) for 24 h. Seeds, peel and whole fruits were triturated to powder separately for quantification. One gram of each part was subjected to initial extraction with hexane in a ratio of 1:5 (*w/v*; three replicates) and then acetonitrile in a ratio of 1:10 (*w/v*; five replicates), with ultrasonication (Unique, Indaiatuba, Brazil) for 30 min. The extracts were filtered and evaporated to dryness in a rotary evaporator at 40 °C (Fisatom, São Paulo, Brazil) and stored at −20 °C until they were analysed by HPLC (Shimadzu Corp. Kyoto, Japan) The yields of hexane and acetonitrile extracts obtained from the seeds, peel and whole fruits of *C. frutescens* were 176 mg of hexane and 38 mg of acetonitrile in the seed extract, 306 mg of hexane and 58 mg of acetonitrile in the peel extract, and 318 mg of hexane and 46 mg of acetonitrile in the whole fruit extract.

### 3.3. Isolation of Compounds from Pimenta malagueta

The compounds capsaicin, dihydrocapsaicin and chrysoeriol used at this work were obtained in our previous study [19].

### 3.4. Quantification of Capsaicin, Dihydrocapsaicin and Chrysoeriol Content

Capsaicin, dihydrocapsaicin and chrysoeriol were quantified by HPLC-DAD according to Collins *et al.* [25] with some modifications. The calibration curve was generated with standard solutions of 31.2, 62.5, 125, 250 and 500 µg mL^−1^ of capsaicin, dihydrocapsaicin and chrysoeriol. The quantitative analyses were performed in triplicate at 290 nm for each sample.

### 3.5. Determination of Total Phenolic Content

The determination of total phenolic content of the *C. frutescens* extracts was performed by the spectrophotometric method of Folin-Ciocalteu [33] with modifications, using gallic acid as the standard.

### 3.6. DPPH Free Radical Scavenging Assay

Free radical scavenging activity of the samples was determined using the DPPH spectrophotometric method according to Silva *et al.* [34].

### 3.7. ABTS Radical Cation Assay

This test involves the generation of the chromophore ABTS^•+^ through the oxidation of ABTS [2,2'-azinobis-(3-ethyl-benzothialoline-6-sulfonic acid)] with potassium persulfate and was performed according to Re *et al.* [35] with modifications. Trolox was used as the positive control.

3.8. β-Carotene Bleaching Test

Antioxidant activity was determined using the β-carotene bleaching test according to Bamoniri *et al*. [29] with modifications. Trolox (16 µg mL^−1^) was used as a standard antioxidant. Absorbance of the samples was taken at zero time, and the measurement of absorbance was continued for 120 min. The antioxidant capacity was expressed as a percentage inhibition of oxidation.

### 3.9. Antimicrobial Assay

#### 3.9.1. Microbial Strains

The microbial strains used belong to Gram positive bacteria (*Staphylococcus aureus* UFPEDA02*, Enterococcus faecalis* ATCC6057, and *Bacillus subtilis* UFPEDA86), Gram negative bacteria (*Escherichia coli* ATCC25922, *Klebsiella pneumoniae* ATCC29665, and *Pseudomonas aeruginosa* UFPEDA416*)* and yeasts (*Candida albicans* UFPEDA1007). The samples were acquired from the Antibiotics Department of the University Federal of Pernambuco, Recife, Brazil.

#### 3.9.2. Determination of Minimal Inhibitory Concentration (MIC)

The broth micro-dilution assay was performed according to CLSI reference methods M7-A6 for bacteria [36] and M27-A3 for yeasts [37]. Ninety-six-well microplates were used to obtain the MIC value of capsaicin, dihydrocapsaicin and chrysoeriol isolated from *C. frutescens* against microorganisms. The concentrations of compounds diluted in DMSO ranged from 0.03 to 100 µg mL^−1^.

The MIC was determined by measuring each well with a microplate reader (ASYS UVM 340, Cambridge, UK). The MIC was defined as the lowest sample concentration that inhibited bacterial growth compared with the optical density of the controls. Chloramphenicol (50 µg mL^−1^) was used as a positive control for all of the strains, and itraconazole (25 µg mL^−1^) was used as a positive control for the yeast.

### 3.10. Statistical Analysis

All samples were analysed in triplicate, and the results were pooled and expressed as the means ± standard error. Statistical analyses were performed with GraphPad Prism version 5.0 (GraphPad Software Inc., San Diego CA, USA). The anti-free radical activity was determined using a linear regression analysis with a confidence interval of 95% (*p* < 0.05). The results were expressed as the EC_50_ ± SEM, which represents the concentration of the sample necessary to reduce the absorbance of DPPH or ABTS^•+^ by 50% compared with the negative control. A one-way analysis of variance (ANOVA) and Tukey’s test were used to evaluate the differences of the means between groups (*p* < 0.05).

## 4. Conclusions

Acetonitrile produced the highest amount of extracted compounds. The whole fruit acetonitrile extract had the highest phenolic content and the best antioxidant activity among the extracts. Regarding the isolated compounds, both capsaicin and dihydrocapsaicin showed low EC_50_ values and high antioxidant activity percentages. On the other hand, we found the best antimicrobial activity against the bacteria tested with the flavonoid chrysoeriol. These findings suggest that should be performed future investigations in order to identify other possible biological and industrial applications.
